# Functional and Phylogenetic Relatedness in Temporary Wetland Invertebrates: Current Macroecological Patterns and Implications for Future Climatic Change Scenarios

**DOI:** 10.1371/journal.pone.0081739

**Published:** 2013-11-28

**Authors:** Albert Ruhí, Dani Boix, Stéphanie Gascón, Jordi Sala, Darold P. Batzer

**Affiliations:** 1 Institute of Aquatic Ecology, University of Girona, Girona, Catalonia, Spain; 2 Catalan Institute for Water Research, Girona, Catalonia, Spain; 3 Department of Entomology, University of Georgia, Athens, Georgia, United States of America; Consiglio Nazionale delle Ricerche (CNR), Italy

## Abstract

In freshwater ecosystems, species compositions are known to be determined hierarchically by large to small‑scale environmental factors, based on the biological traits of the organisms. However, in ephemeral habitats this heuristic framework remains largely untested. Although temporary wetland faunas are constrained by a local filter (i.e., desiccation), we propose its magnitude may still depend on large-scale climate characteristics. If this is true, climate should be related to the degree of functional and taxonomic relatedness of invertebrate communities inhabiting seasonal wetlands. We tested this hypothesis in two ways. First, based on 52 biological traits for invertebrates, we conducted a case study to explore functional trends among temperate seasonal wetlands differing in the harshness (i.e., dryness) of their dry season. After finding evidence of trait filtering, we addressed whether it could be generalized across a broader climatic scale. To this end, a meta-analysis (225 seasonal wetlands spread across broad climatic categories: Arid, Temperate, and Cold) allowed us to identify whether an equivalent climate-dependent pattern of trait richness was consistent between the Nearctic and the Western Palearctic. Functional overlap of invertebrates increased from mild (i.e., Temperate) to harsher climates (i.e., Arid and Cold), and phylogenetic clustering (using taxonomy as a surrogate) was highest in Arid and lowest in Temperate wetlands. We show that, (i) as has been described in streams, higher relatedness than would be expected by chance is generally observed in seasonal wetland invertebrate communities; and (ii) this relatedness is not constant but climate-dependent, with the climate under which a given seasonal wetland is located determining the functional overlap and the phylogenetic clustering of the community. Finally, using a space-for-time substitution approach we suggest our results may anticipate how the invertebrate biodiversity embedded in these vulnerable and often overlooked ecosystems will be affected by long-term climate change.

## Introduction

Several processes operating at varying spatiotemporal scales are known to determine community assembly, a crucial aspect of community ecology [1–3]. Recent theory proposes that first, regional pools are constrained by historical processes (including evolution); second, a subset of the regional pool (limited by chance and dispersal limitation) is available for colonization of particular sites; and finally, environmental (abiotic) filters and biotic interactions impose mutual feedbacks in local communities, ultimately determining their compositions (see review in [4]). This theory, developed mostly for plant communities, is fundamental to conceptual ecology. However, in contrast to plants, some faunal groups such as aquatic invertebrates are typically limited minimally by dispersal capabilities or by biotic interactions [5,6]. In fact, the study of abiotic filters alone has proven extremely useful to improve the mechanistic understanding of invertebrate compositions in streams and wetlands. Poff [7] proposed that large-scale environmental factors, including climatic characteristics, would first select organisms that have matching biological traits, and then these sets would be subsequently sorted by smaller-scale factors. This heuristic framework of landscape filters and species traits has been validated extensively in streams [8–10] and also to some extent in wetlands [11–13]. However, it remains largely untested in ephemeral freshwater habitats.

Ephemeral habitats share a major “local” constraint (i.e., desiccation) that has been identified as being central in determining local compositions (reviewed in 6,14,15). However, some previous works suggest that the magnitude of this control may still covary with larger-scale factors such as climate. For instance, Wissinger [16] proposed that an important difference between eastern and western North-American seasonal wetland faunas is that the dry phase is harsher for invertebrates in the arid west than in the more humid east, with this gradient partly explaining trait composition differences between these regions. Moreover, a recent study on bryophyte, macrophyte, macroinvertebrate and amphibian assemblages of NE Iberian temporary wetlands also found that climate harshness (“mild” vs. “harsh” dry phase wetlands) determined the composition of local assemblages, with climate-dependent patterns prevailing over climate-independent ones [17]. If this pattern is consistent, trait filtering by climate should manifest not only when comparing “mild” vs. “harsh” dry phase wetlands within the same continent, but also between areas differing in their histories (e.g., wetlands in different ecozones, thus supporting unrelated species pools). Furthermore, other climate-dependent aspects of the hydroperiod such as winter freezing should cause analogous effects: north-latitude and high-elevation wetlands may experience a harsh phase because liquid water is not present during portions of the winter, even if not “dry” [16]. 

Due to a lack of long-term biomonitoring efforts for freshwater species (but see 18–21), ‘space-for-time’ substitution approaches may be of particular utility in freshwater habitats. Such studies examine species distributions under current climatic conditions, and use correlations from different bioclimatic envelopes to predict shifts in projected future climate scenarios [22]. Therefore, studying how climate characteristics currently determine functional and phylogenetic aspects of invertebrate communities may suggest how and to which extent these communities will be impaired by climate change in the future. Similar approaches have been previously developed for stream macroinvertebrates (e.g. comparing stream communities from the Mediterranean Basin and the temperate Europe [10]), but to our knowledge never for temporary wetland macroinvertebrates.

**Figure 1:**
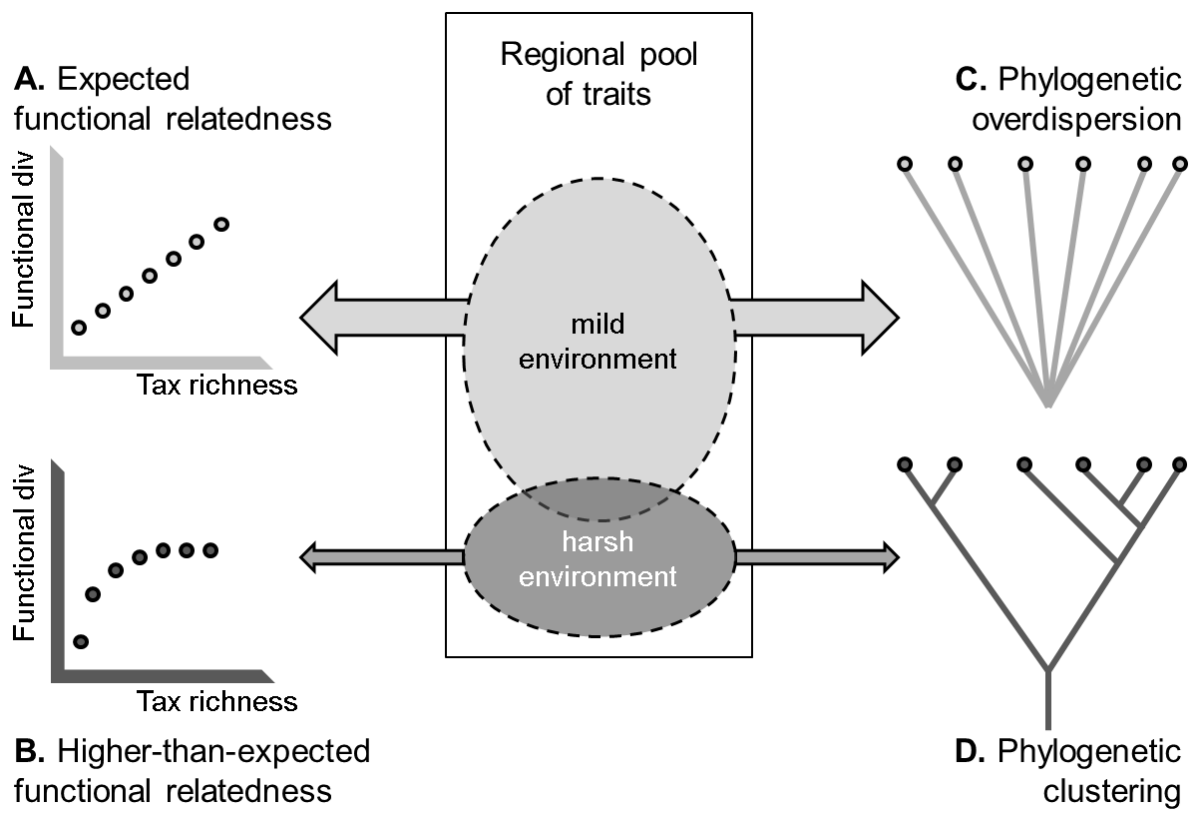
Conceptual framework of trait filtering by climate and expected associated patterns of functional (A, B) and phylogenetic relatedness (C, D). (A) Shows no functional overlap (i.e., expected functional relatedness); (B) shows functional overlap (i.e., higher-than-expected functional relatedness); (C) shows phylogenetic overdispersion; (D) shows phylogenetic clustering.

We propose that certain environmental filters may prevent species with (or without) particular traits from occurring in local communities. Therefore, in these cases functional diversity may be lower than expected by chance, indicating that assemblages contain species with similar functional traits. It has been observed that avian assemblages are filtered by trait suitabilities [23]. We suggest that in wetlands, regardless of the stressor (drying or freezing), filtering by climate should also determine the functional relatedness of local communities. In particular, we suggest that communities inhabiting relatively more “filtered” ecosystems may show higher levels of functional overlap, since species forming these communities will probably share more traits than would be expected by chance. As a consequence, functional diversity would be related to taxonomic richness in mild environments (Figure 1A) but not necessarily in “filtered” ones (Figure 1B). In the latter case, due to trait filtering, functional diversity would not achieve values as high as would be expected under a null assumption of random assembly. On the other hand, because biological traits rely on phylogeny, functionally similar species are likely to be phylogenetically related [24]. Thus, following the same rationale, we propose that communities in mild environments will be relatively more prone to phylogenetic overdispersion (Figure 1C) than communities inhabiting “filtered” habitats. In turn, filtered communities in harsh environments would tend to show greater phylogenetic clustering (Figure 1D), since their community members would be, on average, phylogenetically closer than would be expected by chance due to the filtering for traits suitable for tolerating the increased harshness. 

We tested this proposed framework in two ways. First, we explored how climate is related to trait richness and functional diversity of local communities using a case study in two unconnected regions at similar latitudes, but with differing harshness during the dry season (*Harsh* vs. *Mild Summer Temperate* wetlands). We hypothesized that: (i) higher trait richness would occur in the *Mild* than the *Harsh Summer* region; (ii) trait composition would differ between regions, due to biological traits that permit taxa to persist through the dry season being dominant in *Harsh Summer* conditions; and (iii) functional diversity would be predicted by taxonomic richness in *Mild* but not in *Harsh Summer* wetlands, with the latter presenting lower functional diversity values than would be expected if communities were randomly assembled. Next, we tested whether observations in the case study could be generalized across broader climatic scales. To this end, we supplemented the case study with additional seasonal wetlands (≥100 in each ecozone, Nearctic and Western Palearctic), spread across broad climatic categories (*Arid*, *Temperate*, and *Cold* climates). We hypothesized that in both ecozones: (iv) significant differences in trait richness and composition among climates would be observed; (v) functional overlap would increase when moving from less (i.e., *Temperate* climates) to more climate-filtered wetlands (i.e., *Arid* and *Cold* climates), due to the harsher conditions during dry/frozen periods; and (vi) this functional relatedness trend would be associated to a phylogenetic relatedness pattern, namely phylogenetic clustering increasing from *Temperate* to both *Arid* and *Cold* climates.

## Materials and Methods

### Ethics Statement

The field studies did not involve endangered or protected invertebrate species. Therefore, we did not require ethical approval to conduct the current study. In Georgia (USA) no specific permissions were required to access study sites, since wetlands were located in public, state-owned lands. AR received permission from the Ministry of the Environment of the Government of Catalonia (Permit #SF/317) and “Base Militar General Álvarez de Castro” to access study sites in Catalonia. Permissions from private owners were received to access locations in Sardinia.

### Study design

**Figure 2:**
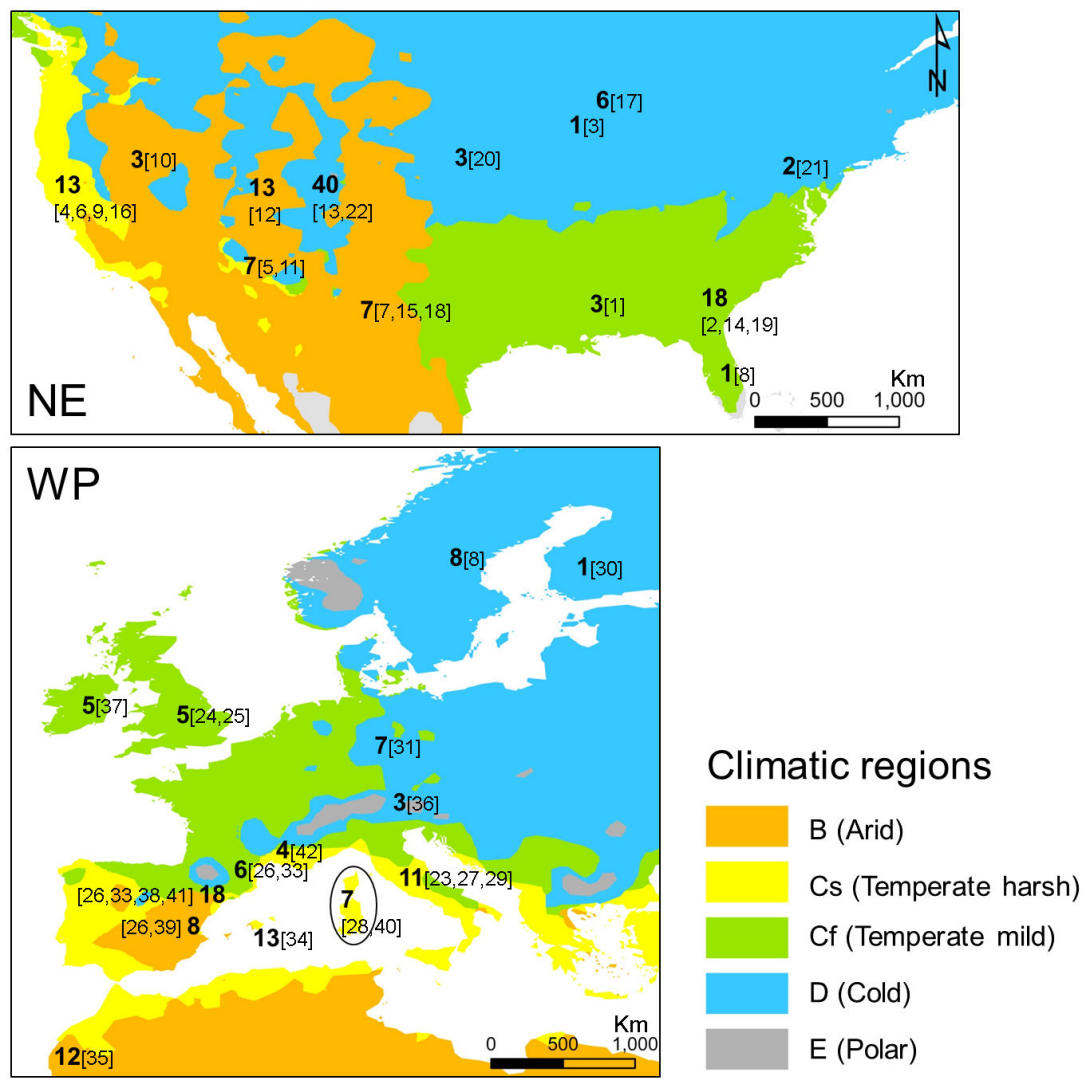
Map showing the number and approximate location of the 225 seasonal wetlands considered in the meta-analysis (bolded) and the corresponding source in Table A in File S1 (in brackets). The climatic regions according to Köppen classification are shown. For details about each study, see Table A in File S1. NE = Nearctic, WP = Western Palearctic.

Two distinct macroinvertebrate data sets were used: (1) a case-study from 16 seasonal wetlands occurring in temperate climates; and (2) 225 seasonal wetlands, combining data from this study with literature data (Figure 2, Table A in File S1).

### Case-study areas and sampling procedure

Two regions were selected in the temperate zone, with two wetland areas in each and four wetlands in each area. According to the climate classification system of Köppen [25] and more recent updates [26,27], one region fell under the Cs category (Warm temperate climate with dry summer; hereafter *Harsh Summer Temperate* climate) and the other under the Cf category (Warm temperate climate, fully humid; hereafter *Mild Summer Temperate* climate). The *Mild Summer Temperate* wetlands were found in the Southeastern USA (Georgia), and the *Harsh Summer Temperate* wetlands were in the Mediterranean basin (Catalonia and Sardinia). All 16 temporary wetlands were minimally affected by humans, and selection of sites aimed to encompass a wide range of natural variability of each area, mainly regarding size and hydroperiod length (Table 1). These temporary wetlands were followed over a complete inundation-drying cycle, and were sampled three times during the hydroperiod: at the beginning (soon after filling), middle, and end (as they dried). At each visit, water conductivities were measured using hand-held meters, and water levels were measured using a graduated gauge. Aquatic macrofauna were sampled using a 20-cm-diameter dip-net (mesh size 250 μm). The sampling procedure was based on 20 dip-net sweeps in rapid sequence spanning all different mesohabitats. Invertebrate fauna samples were subsequently sorted and identified in the laboratory. Identification was mostly to genus, except for turbellarians, oligochaetes, water mites, and chironomids, which were left at family level. Cnidaria (Leptolida), Platyhelminthes (Turbellaria), Annelida (Oligochaeta, Hirudinea), Mollusca (Bivalvia, Gastropoda) and Arthropoda (Branchiopoda, Malacostraca, Arachnida, Insecta) were the phyla and classes considered in this study.

**Table 1:** Details of the 16 temporary wetlands selected for the case study.

**Climate, location**	**Site**	**Latitude**	**Longitude**	**Year**	**Max. surface (m^2^)**	**Altitude (m.a.s.l.)**	**Max. hydroperiod (months)**	**Max depth (cm)**	**Conductivity (μS.cm^-1^, mean ± SD)**
Cf, Georgia (USA)	BB	32°50'33"N	81°30'33"W	2011	13060	29	≈ 7	60	38.00 ± 21.5
	FB	32°50'37"N	81°30'21"W	2011	5260	27	≈ 5	40	108.60 ± 142.1
	GB	32°50'26"N	81°30'21"W	2011	37410	26	≈ 7	110	65.67 ± 49.5
	PB	32°50'42"N	81°30'02"W	2011	12390	27	≈ 7	50	41.67 ± 6.4
Cf, Georgia (USA)	OX	33°45'45"N	83°16'34"W	2011	1560	137	≈ 6	60	97.33 ± 49.9
	S1	33°45'06"N	83°17'08"W	2011	1510	139	≈ 7	50	77.33 ± 22.1
	S3	33°45'48"N	83°16'41"W	2011	2450	137	≈ 7	55	73.67 ± 17.4
	WB	33°46'04"N	83°16'50"W	2011	10600	138	≈ 8	100	122.67 ± 55.8
Cs, Catalonia (Spain)	MAR	42°23'52"N	2°55'44"E	2009	6590	145	≈ 6	50	210.57 ± 21.5
	SER	42°22'51"N	2°56'32"E	2009	4180	121	≈ 5	50	200.33 ± 34.5
	TOG	42°22'53"N	2°57'30"E	2009	34860	143	≈ 9	91	308.67 ± 63.4
	TOP	42°22'45"N	2°57'32"E	2009	13000	142	≈ 7	98	353.67 ± 129.5
Cs, Sardinia (Italy)	LOE	40°34'05"N	9°19'01"E	2007	930	798	≈ 6	16	176.67 ± 15.9
	MON	40°21'44"N	8°20'30"E	2007	590	436	≈ 5	23	120.25 ± 31.6
	PUD	40°20'37"N	8°54'10"E	2007	720	1033	≈ 5	11	363.87 ± 290.5
	SCA	40°13'19"N	8°41'08"E	2007	820	722	≈ 7	16	125.00 ± 27.5

### Literature data for the meta-analysis

Previously published studies supplemented with unpublished data from some authors provided additional lists of taxa originating from three Köppen-Geiger climatic regions in each ecozone (*Arid*, B; *Temperate*, C; and *Cold*, D). To qualify for inclusion, sites within each study were required to: (i) come from non-permanent freshwater lentic systems; (ii) focus on natural systems (i.e., artificial and constructed wetlands were not included); (iii) contain raw data for individual wetlands (i.e., not composite or regional lists); (iv) deal with the whole macrofaunal community (i.e., with sampling methods able to capture all phyla considered); and (v) identify invertebrates mostly to the genus level. After this selection of sites, the data extraction procedure aimed to balance, whenever possible, the amount of spatiotemporal variability and effort considered by studies. When data from several sampling protocols or microhabitats were compared, all samples per visit were aggregated. When several samples through time were collected independently from a particular site, and these samples occurred throughout one hydroperiod, we considered only up to three visits (corresponding to the beginning, middle and final phases). However, if they corresponded to single samples across several hydroperiods, two random samples were selected to account for temporal variability. Both details of the sampling protocol and number of visits were annotated for each wetland, to later assess the extent to which the methodological disparity could affect composition analyses. The selection procedure provided a matrix of 209 sites (109 in the Nearctic, hereafter NE; 100 in the Western Palearctic, hereafter WP) that were added to the 16 sites from the case study (see Figure 2 and Table A in File S1 for details). This resulted in a 491 taxa × 225-site matrix (117 in NE, 108 in WP), ranging from 15 to 59 wetlands per climate at each ecozone.

### Biological traits data base

In order to assign biological traits to taxa, we first aimed at assembling a comparable traits data base across ecozones. For the WP, Tachet et al. [28] was the reference trait database selected, and for the NE we used the recent data base from the U.S. Environmental Protection Agency [29], which combines several existing trait databases into a unified free-accessible online database. In order to achieve comparable data bases, the particular traits considered under each category, and the corresponding ranges of each (e.g., values and meaning of traits) were compared, and only biological categories and traits considered by both data bases were taken into account. This procedure caused deletion of some traits and the creation of some new trait groups (see Table 2 for the traits considered; Table B in File S1 for details on the merging procedures). We ended up with a genus × traits binary database, showing each of the 52 biological traits (present/absent) for the taxa detected in the study (hereafter, *traits database*).

**Table 2:** Functional categories and biological traits considered in this study.

**Functional category**	**Biological trait**	**Functional category (cont.)**	**Biological trait (cont.)**
**Body size**	large	**Microhabitat (cont.)**	gravel
	medium		sand
	small		silt
**Respiration**	gills		macrophytes
	plastron, spiracle		microphytes
	integument		detritus/litter
**Reproduction**	isolated eggs, free	**Exit temporarily**	absent
	isolated eggs, cemented		present
	clutches, cemented or fixed	**Number of aquatic stages**	1 (larva/nymph only)
	clutches, free		2 (egg, larva/nymph)
	clutches, in vegetation		3 (egg, larvae/nymph, adult; or egg, larva, pupa)
	clutches, terrestrial		4 (egg, larva, pupa, adult)
**Feeding mode**	filter-feeder	**Voltinism**	< 1
	collector		1
	scraper		> 1
	shredder	**Trophic preferendum**	oligotrophic
	piercer		mesotrophic
	predator		eutrophic
	parasite	**pH preferendum**	acidic
**Habitat**	attached/fixed		normal
	burrower		alkaline
	skater	**Salinity preferendum**	fresh waters
	swimmer		brackish waters
	interstitial	**Temperature preferendum**	psychrophilic
	other		thermophilic
**Microhabitat**	flags/boulders/cobbles/pebbles		eurythermic

### Statistical analyses

#### Functional trends in temperate seasonal wetlands

In order to assess functional differences between Harsh and Mild Summer *Temperate* wetlands, we developed a biological traits abundance matrix. Abundances of genera at each site in the case study were combined with the *traits database*, generating a 16 sites × 52 biological traits abundance matrix. We then conducted three different analyses. First, we compared trait and taxonomic richness between regions (*Harsh* vs. *Mild Summer Temperate*) using an ANOVA. Second, trait composition was assessed using ANOSIM (analysis of similarities) and SIMPER (similarity percentages) analyses. In order to compare trait composition between regions, we used a 2-factor layout ANOSIM with *region* as the main factor (*Harsh* vs. *Mild*), with particular sites nested within each region (abundance data log-transformed, Bray-Curtis index as the similarity metric, 999 permutations). ANOSIM delivers a global R that ranges from 0 to 1 and assigns a *p*-value. When R approaches 0, similarities within groups and between groups are equivalent. When higher values of R are obtained, samples within the group resemble each other more than they do between groups, thus differentiating groups from different levels of the factor. The SIMPER analysis identified the particular traits that most explain functional differences between regions. Third, the TAXDTEST routine [30] was run on the same matrix to test the null hypothesis (H_0_) that the functional composition of invertebrates behaved as though traits in local communities were assembled at random from the regional traits pool (model 3 in [31]). This procedure plots the observed functional diversity values against a probability funnel that accounts for taxonomic richness. Functional diversity was approached as average functional distinctness (Χ^+^), a functional relatedness measure based on trait similarities (simple matching coefficient on trait presence-absence data) among species within a community (after [32]). In this sense, samples falling above the 95% probability funnel would reject the null hypothesis (H_0_) by exhibiting higher-than-expected functional diversity values (H_A1_), whereas those falling below the 95% threshold would reject the null hypothesis by presenting a lower functional diversity than would be expected according to their genus richness (H_A2_).

#### Global meta-analysis on functional and phylogenetic relatedness

Given that data were collected using different methodologies and devices, and aggregated different numbers of sampling periods, the possible effects of these unbalances on genus composition were assessed. To this end, we performed two ANOSIM tests, in order to test for differences among (i) the different sampling methodologies and devices used (dip-net, core, kick, Hess, Surber, and their combinations; grouped into 12 categories), and (ii) the varying numbers of visits per site conducted (from 1 to > 10, grouped into 5 categories). In both tests the 2-factor layout was selected, with factors *sampling procedure* or *number of visits* nested within the *ecozone* factor (Bray-Curtis index as the similarity metric, 999 permutations). If significant compositional differences were explained by differences in the sampling procedure or the number of visits, a pre-selection of sites would have been required to homogenize the data set. However, ANOSIM tests did not detect compositional differences associated with the sampling method used (R = -0.576, *p* = 0.994) or the number of visits (R = 0.070, *p* = 0.356). Thus, all 225 sites (hereafter, *taxa matrix*) were retained in subsequent analyses. The *taxa matrix* was combined with the *traits database*, delivering a 225 sites × 52 biological traits matrix (hereafter, *traits matrix*). As a preliminary exploration, taxonomic and trait composition patterns were visualized by means of a non-parametric Multi-Dimensional Scaling (Bray-Curtis similarity coefficient).

We aimed at investigating (i) functional relatedness and (ii) phylogenetic relatedness patterns on these global data sets. Regarding (i) functional relatedness, we first tested for significant structure in the *traits matrix* by means of SIMPROF (similarity profile), a permutation-based routine [30]. This test calculates Bray-Curtis composition similarities from a group of samples, plotting them against their rank. Then, the similarity profile is compared with that expected under the null hypothesis of no meaningful structure within the matrix. The existence of significant structure (high *Pi* values and *p* < 0.05) can be used as an objective basis to further explore the impact of signals on this structure. Subsequently, an ANOSIM tested for significant trait composition differences between ecozones, and among climates within each *ecozone* (2-factor layout: *climate* nested within *ecozone* factor). We then performed a test of model effects using a Generalized Linear Model (*trait richness* as the response variable and two fixed effects: *climate* nested within *ecozone*). Finally, functional overlap was addressed with a niche overlap model, which tests for variation among species in niche utilization. In our case, rows were genera and columns were the biological traits potentially present for each of the genera. A model was run for each of the 225 wetlands, with biological trait values (presence/absence) randomly shuffled among taxa present at each wetland (Pianka niche overlap index, 999 iterations). The null hypothesis (H_0_) that the observed overlap does not significantly differ from the expected overlap was compared to two alternative hypotheses; (H_A1_) lower-than-expected overlap; and (H_A2_) higher-than-expected overlap. 

Additionally, we used (ii) taxonomic relatedness as a proxy for phylogenetic relatedness, because phylogenetic information was not immediately available for all the taxa that occurred. Other studies concluded that the use of taxonomic ranks as surrogates of phyletic distances only slightly affects the estimates of phylogenetic distinctness between plots [33]. We selected the *taxa matrix* and used the SPECDIST routine to generate a taxonomic distance matrix for each *ecozone*, with distance values representing length of path connecting genus pairs, traced through a taxonomic tree that considered six taxonomic levels from genus to domain (genus, family, order, class, phylum, domain) [34]. We then calculated taxonomic relatedness based on Average Taxonomic Distinctness, Delta+ [34]. We performed a test of model effects using a Generalized Linear Model with *taxonomic relatedness* (*Delta+*) as the response variable and two fixed effects: *climate* nested within *ecozone*. As for the case study, the TAXDTEST routine [30] allowed us to test the null hypothesis that presence of a genus represented a random selection from the pool (model 3 in [31]). Hence, this procedure permitted a determination of whether taxa in a sample present a higher- or lower-than-expected taxonomic relatedness (Delta+). Again, if a sample falls inside the 95% probability funnel, the null hypothesis (H_0_) cannot be refuted, whereas if it falls either above (H_A1_, overdispersion) or below (H_A2_, clustering), it is rejected. 

ANOSIM, MDS, RELATE, TAXDTEST, SIMPER and SIMPROF analyses were performed with PRIMER-E v.6.1.11 & PERMANOVA + v.1.0.1 (PRIMER-E Ltd.), functional overlap was assessed by means of EcoSim Professional (Acquired Intelligence Inc.), and ANOVA and Generalized Linear Models were conducted with the software package PASW v.18 (SPSS).

## Results

### Functional trends in temperate seasonal wetlands

Temporary wetlands in the *Harsh Summer Temperate* region presented significantly lower trait richness values than those in the *Mild Summer Temperate* region (*Harsh*: S_Traits_ [mean ± SE] = 46.25 ± 0.79, *Mild*: S_Traits_ = 49.00 ± 0.40; *F*_1,45_ = 9.415, *p* < 0.005). However, no significant differences in taxonomic richness were found between regions (*Harsh*: S_Tax_ [mean ± SE] = 14.75 ± 1.46, *Mild*: S_Tax_ = 16.00 ± 1.09; *F*_1,45_ = 0.464, *p* > 0.1).

Regarding trait composition, ANOSIM tests showed significant differences between regions (R = 0.539, *p* < 0.001), with composition being consistent among the eight wetlands within each region (R = - 0.052, *p* > 0.5). A subset of 11 out of 52 biological traits explained >50% of the functional dissimilarity between regions, with five particular traits associated with *Harsh Summer Temperate* wetlands and six traits with *Mild Summer Temperate* ones (details of the SIMPER analysis are shown in Table 3). 

**Table 3:** Results of the SIMPER analysis.

**Biological trait**	**Temperate region dominance**	**Av. Diss**	**Diss/SD**	**Contrib%**	**Cum.%**
Number of aquatic stages: 4	Mild Summer (Cf)	2.00	1.11	6.16	6.16
Bodysize: large	Harsh Summer (Cs)	1.69	2.02	5.19	11.35
Reproduction: isolated eggs, free	Mild Summer (Cf)	1.66	1.33	5.1	16.45
Feeding mode: parasite	Mild Summer (Cf)	1.61	1.28	4.97	21.42
Habitat: skater	Harsh Summer (Cs)	1.58	1.35	4.85	26.27
Reproduction: clutches, terrestrial	Mild Summer (Cf)	1.55	1.35	4.76	31.03
Voltinism: <1	Mild Summer (Cf)	1.47	0.98	4.51	35.54
Reproduction: clutches,free	Harsh Summer (Cs)	1.38	1.69	4.25	39.79
Habitat: interstitial	Harsh Summer (Cs)	1.18	1.43	3.63	43.43
Habitat: attached/fixed	Mild Summer (Cf)	1.17	1.24	3.6	47.02
Habitat: burrower	Harsh Summer (Cs)	1.15	1.64	3.54	50.57

The biological traits contributing more to dissimilarity between *Harsh (Cs*) and *Mild Summer (Cf*)* Temperate* wetlands are shown. Av.Diss = average dissimilarity of each biological trait between climates; Diss/SD = dissimilarity between climates/standard deviation; Contrib% = percentage contribution to dissimilarity between climates; Cum.% = cumulative contribution to dissimilarity between climates.

**Figure 3:**
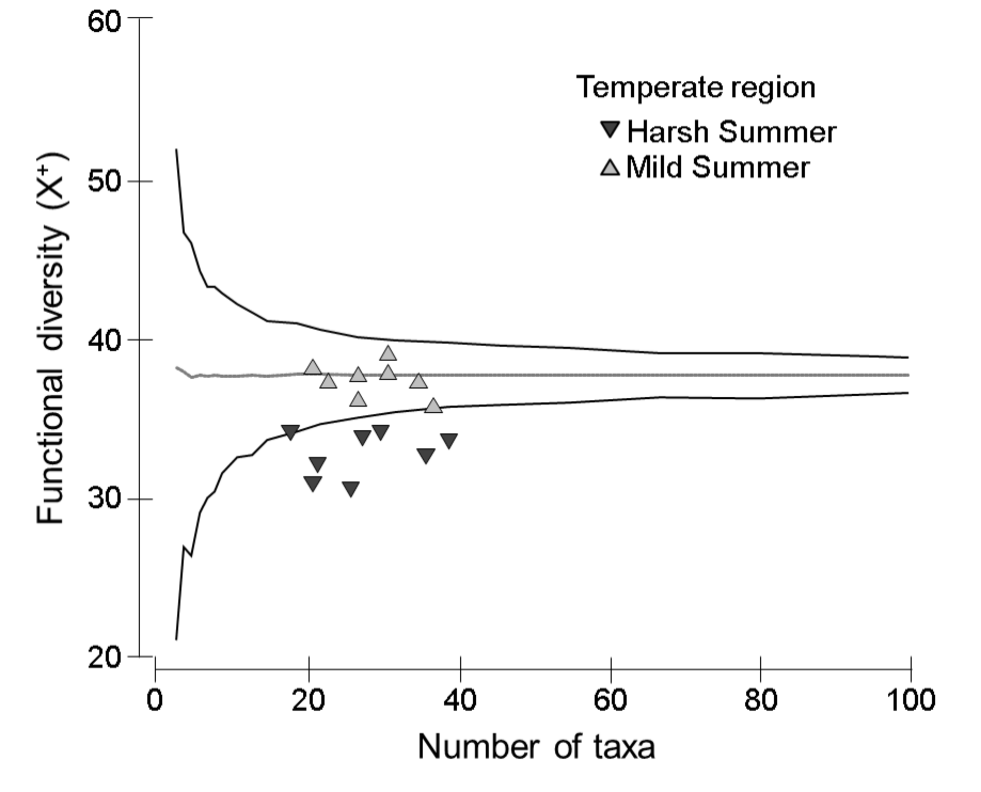
Functional diversity trends vs. taxonomic richness in the Temperate wetlands of the case study The communities observed over a complete hydroperiod in *Harsh* *Summer* *Temperate* wetlands (dark symbols) and *Mild* *Summer* *Temperate* wetlands (light symbols) were plotted against a 95% probability funnel using the TAXDTEST routine. Whereas wetlands within this funnel host communities with traits that could be explained as random draws from the pool (null hypothesis, H_0_), those below the 95% probability threshold present a significantly lower functional diversity than would be expected according to taxonomic richness (H_A2_).

Finally, as hypothesized the functional diversity at a given wetland could be explained as a random draw of biological traits in the *Mild Summer Temperate* region (i.e., H_0_ was not rejected) but not in the *Harsh Summer Temperate* region, where functional diversity values were lower than those predicted by taxonomic richness (i.e., H_A2_ was accepted) (Figure 3).

### Global meta-analysis on functional and taxonomic relatedness

**Figure 4:**
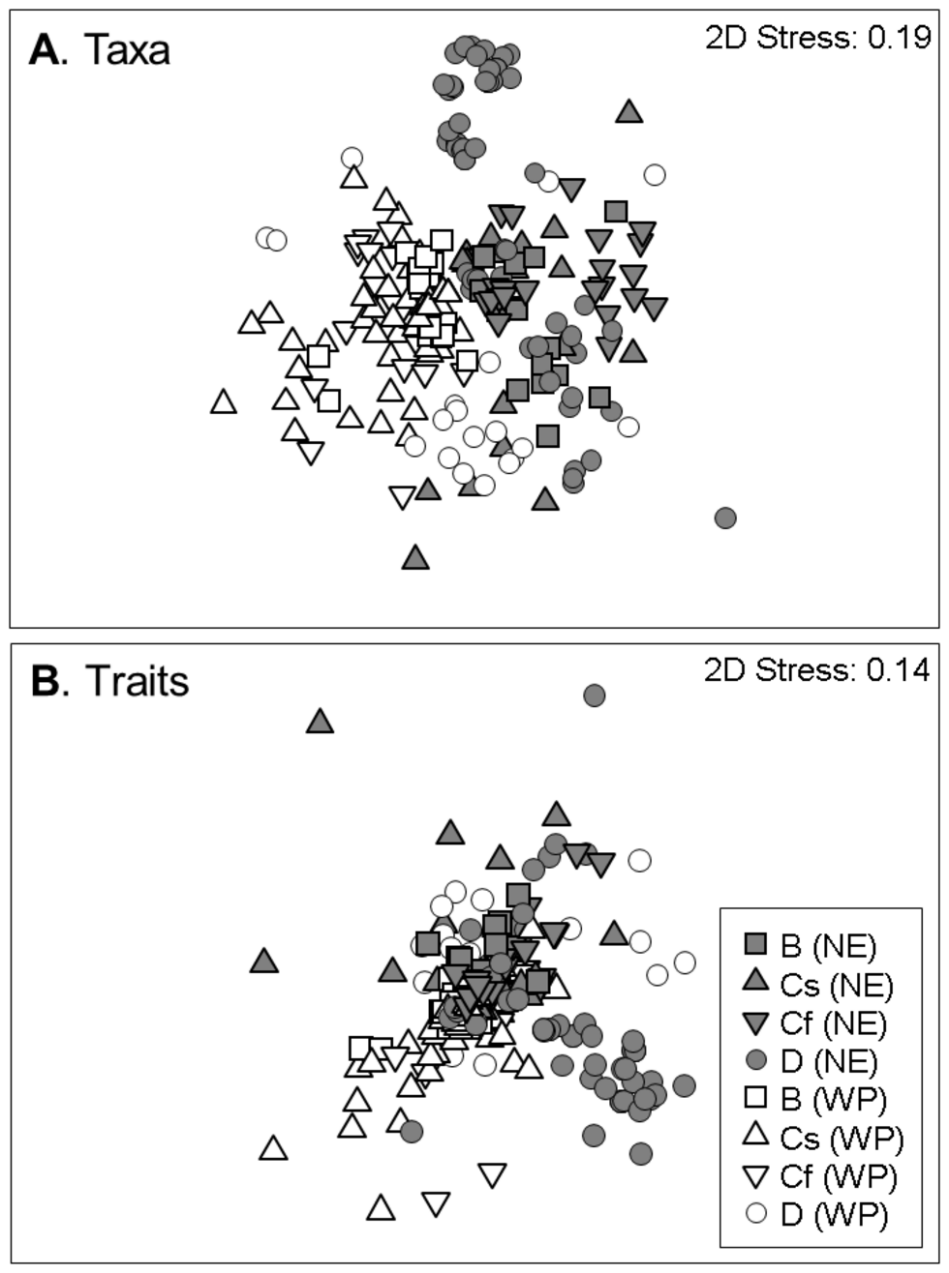
MDS showing sites according to their (A) taxonomic composition and (B) trait composition similarities, classified by climates (Arid, B; *Temperate Harsh Summer*, Cs; *Temperate Mild Summer*, Cf; and Cold climate, D) and ecozones (NE = Nearctic, WP = Western Palearctic).

**Figure 5:**
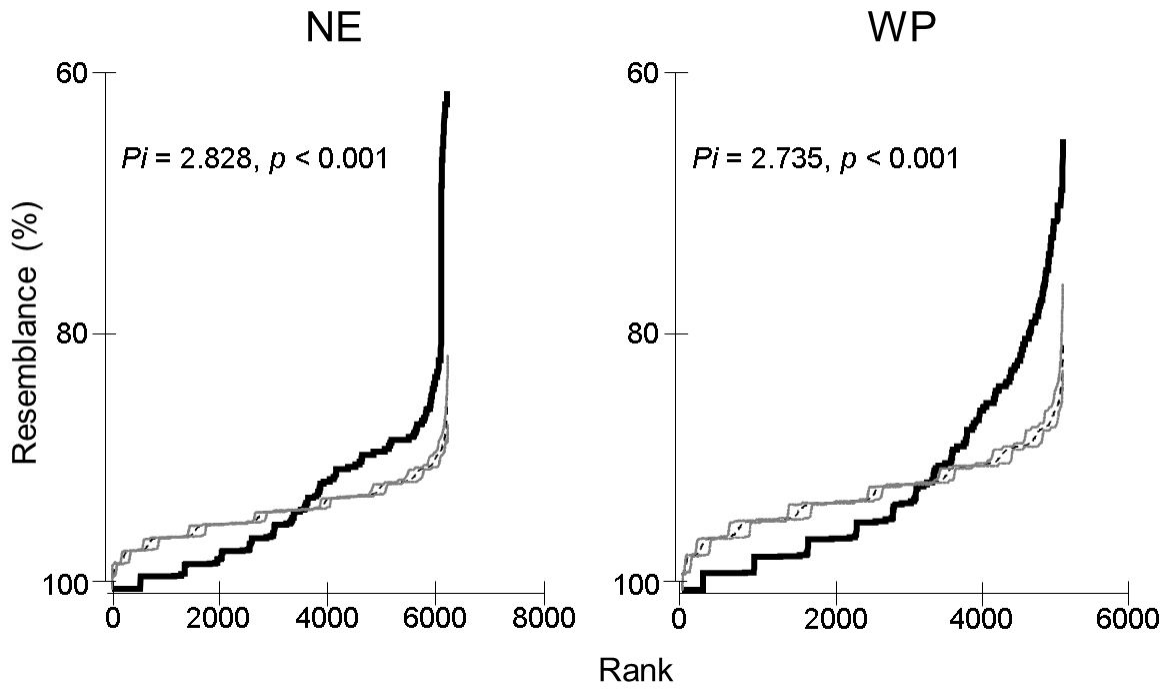
Similarity profiles (SIMPROF) for the traits matrix at each ecozone (NE = Nearctic, WP = Western Palearctic). The profile shows the ordered similarities between sites plotted against their real ranks (black thick continuous line) and permuted data (mean of the 999 permuted matrices in black thin dashed line and 99% confidence intervals in grey lines). In both cases, higher similarities between site pairs were observed less than expected by chance (lower ranks), while the opposite was true for lower similarities (higher ranks). The statistics (Pi) and *p*-values are provided.

**Figure 6:**
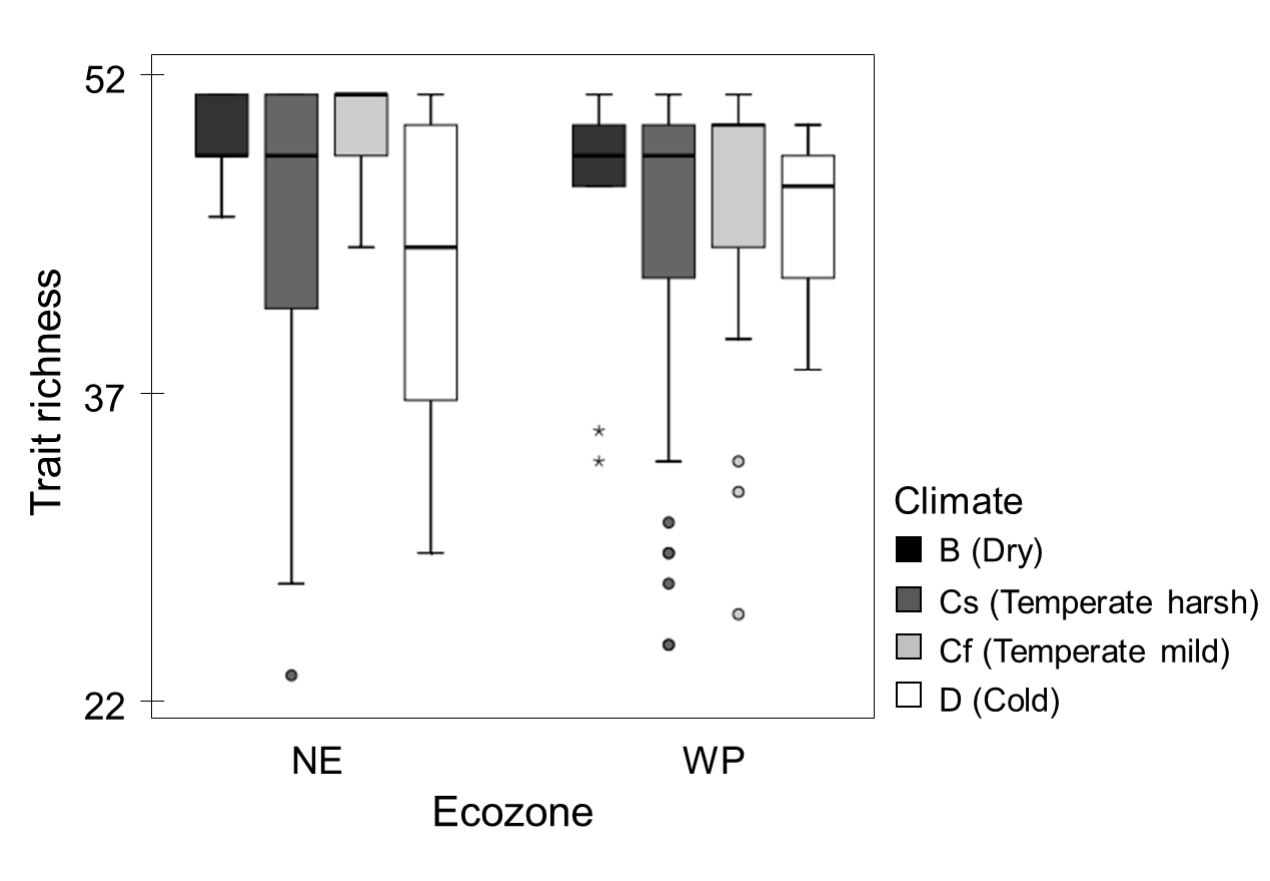
Trait richness trends among climates within each ecozone (NE = Nearctic, WP = Western Palearctic).

When ordered by taxonomic composition similarities, sites were grouped into ecozones (Figure 4A). However, these differences dissipated when plotting sites by trait composition similarities (Figure 4B). Within each ecozone, local trait compositions could not generally be explained as a random draw from the regional pool, neither in the NE nor in the WP (SIMPROF tests, Figure 5). Instead, there was significant structure to be explored. The ANOSIM tests showed that sites grouped into climates, regardless of their ecozone: trait composition differed among climates within each ecozone (ANOSIM, R = 0.155, *p* < 0.001) despite no significant differences observed among ecozones overall (ANOSIM, R = 0.198, *p* > 0.05). Trait richness differed among climates within each ecozone (Wald Chi-Square = 26.037, df = 6, *p* < 0.001) despite no differences between ecozones (Wald Chi-Square = 1.092, df = 1, *p* > 0.1). This parameter peaked under the *Mild Summer Temperate* climate in both ecozones, with values decreasing towards *Arid* and *Cold* climates (Figure 6). 

The observed functional overlap for all 225 wetlands was significantly higher than expected by chance (*p* < 0.05). Hence, the null hypothesis (H_0_) that the observed overlap can be explained as a random draw of traits from the pool was rejected in favor of the functional overlap alternative hypothesis (H_A2_). Mean overlap values per climate showed a consistent trend between ecozones (Table 4): following our predictions, the lowest functional overlap was found in *Mild Summer Temperate* wetlands, and it increased towards both drier (Cs, B) and *Cold* climates (D). The same trend was observed in the simulated run, with overlap values generated by the null model.

**Table 4:** Summary results of the functional overlap tests.

**Ecozone**	**Climate**	**N sites**	**Observed overlap (mean ± SE)**	**Simulated overlap (mean ± SE)**
**NE**	B (Dry)	15	0.472 ± 0.03	0.362 ± 0.02
	Cs (Temperate, Harsh Summer)	21	0.419 ± 0.01	0.308 ± 0.01
	Cf (Temperate, Mild Summer)	22	0.395 ± 0.01	0.303 ± 0.01
	D (Cold)	59	0.433 ± 0.01	0.328 ± 0.01
**WP**	B (Dry)	20	0.564 ± 0.02	0.399 ± 0.01
	Cs (Temperate, Harsh Summer)	53	0.571 ± 0.01	0.393 ± 0.01
	Cf (Temperate, Mild Summer)	16	0.549 ± 0.02	0.392 ± 0.01
	D (Cold)	19	0.585 ± 0.02	0.436 ± 0.02

The observed functional overlap for each of the 225 studied wetlands was significantly higher than expected (p < 0.05). This table summarizes results for wetlands falling under the same climate and ecozone. Observed overlap = niche overlap values averaged across wetlands under the same climate and ecozone; Simulated overlap = niche overlap values simulated by the null models, averaged across wetlands under the same climate and ecozone.

**Figure 7:**
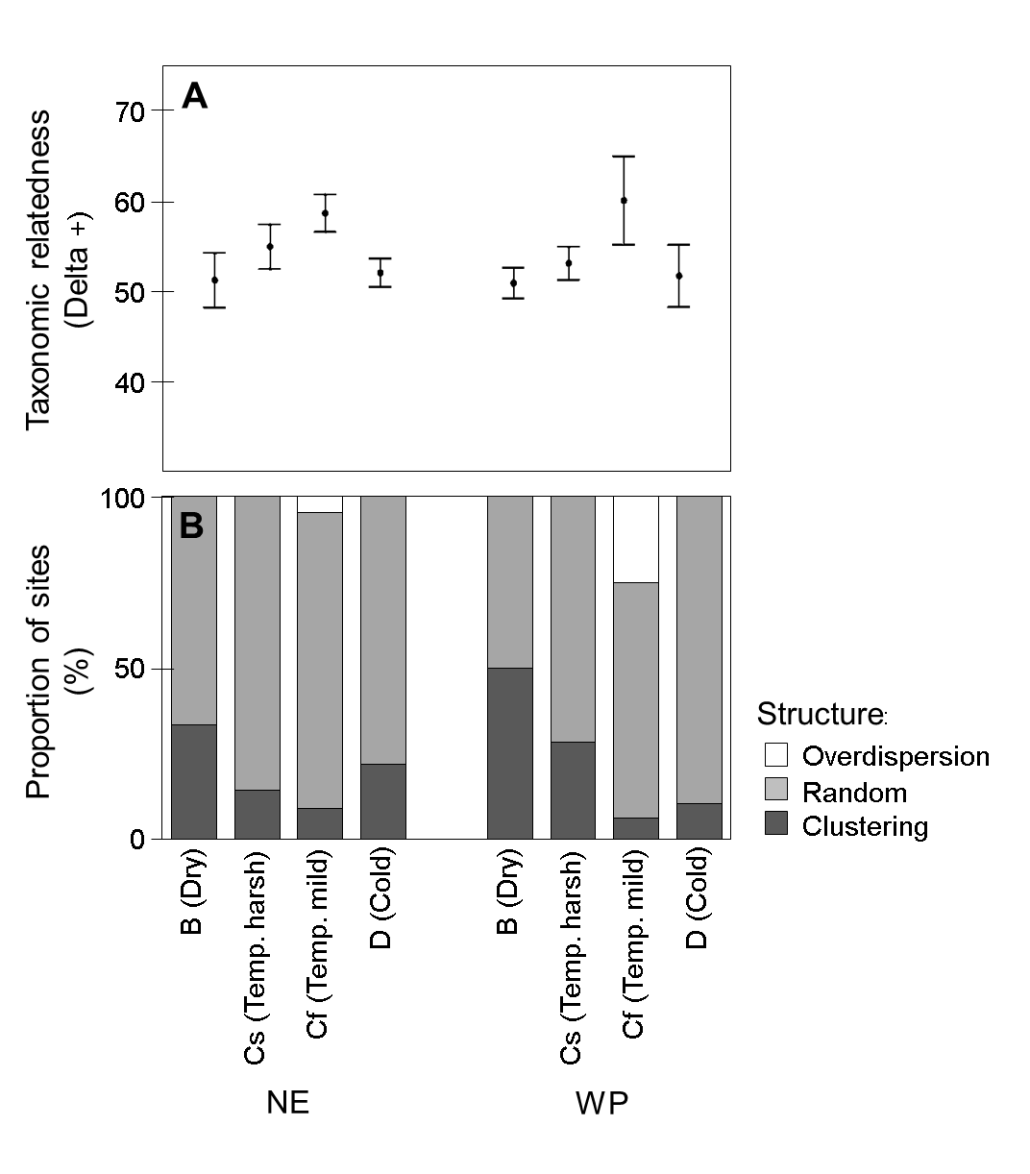
Patterns of taxonomic clustering among climates within each ecozone (NE = Nearctic, WP = Western Palearctic). (A) Mean ± SE values of Delta+, informing about taxonomic relatedness (relatively higher Delta+ values imply assemblages with taxonomically closer members); (B) % of sites presenting lower-than-expected taxonomic relatedness (H_A1,_ overdispersion), expected taxonomic relatedness (H_0_, random clustering) and higher-than-expected taxonomic relatedness (H_A2,_ clustering).

Finally, taxonomic relatedness patterns differed among climates within each ecozone (Wald Chi-Square = 46.449, df = 6, *p* < 0.001), but not across ecozones overall (Wald Chi-Square = 0.090, df = 1, *p* > 0.5). The highest values of Average Taxonomic Distinctness (Delta+) were found in *Mild Summer Temperate* wetlands and decreased towards *Arid* and *Cold* climates in both ecozones, as predicted (Figure 7). Only in the *Mild Summer Temperate* climate did some wetlands exhibit a lower-than-expected taxonomic relatedness (H_A1_, *overdispersion*), with 1 site in the NE (5 % of sites under this climate) and 4 sites in the WP (25 %). On the other hand, only a few cases of higher-than-expected taxonomic relatedness (H_A2_, *clustering*) were found in *Mild Summer Temperate* wetlands, with 2 sites in the NE (9 % of sites under this climate) and 1 site in the WP (6 %). As expected, the number of taxonomically-clustered sites tended to increase when moving towards *Arid* and *Cold* climates, achieving maximum values in the *Arid* climate in both ecozones, with 5 sites in the NE (33 % of wetlands under this climate) and 10 sites in the WP (50 %) (Figure 7).

## Discussion

### The heuristic framework of environmental filters and species traits

This study corroborated the assumption that environmental filtering is a primary influence on community assembly of seasonal wetland invertebrates, as had been previously described for stream invertebrates [7,9,35,36]. Indeed, in the first section of the study we observed the impact of filtering by climate on functional diversity, by comparing macroinvertebrate assemblages inhabiting *Mild* vs. *Harsh Summer Temperate* wetlands. Functional diversity differed according to predictions: despite presenting similar levels of taxonomic richness, these regions differed in the number of traits that were frequently observed. In the case of *Harsh Summer Temperate* wetland communities, this mechanism elicited lower-than-expected values of functional diversity. 

Within this context, although not all trait differences between *Mild* and *Harsh Summer Temperate* could be explained by climatic filtering, the observed patterns for traits explaining the main differences between these climates were certainly expected. For example, the primary selected trait discerning both regions (taxa with all 4 stages aquatic) was more abundant in *Mild* rather than in *Harsh Summer Temperate* wetlands. This result follows Wissinger’s [16] idea that humid seasonal wetlands should support more fully-aquatic invertebrates and fewer cyclic colonizers (i.e., aerial colonizers that migrate between permanent and temporary waters to complete their life cycle, sensu [6]) than drier seasonal wetlands, and vice-versa. For another selected trait (semivoltinism, also dominant in *Mild* rather than *Harsh Summer Temperate* wetlands), the similar result was found between Mediterranean and cold temperate regions when comparing streams [10] or man-made wetlands [12]. Both of these studies had hypothesized a dominance of traits favoring resilience from and resistance against disturbances (e.g., multivoltinism, resistance forms, specialized respiration techniques) in harsh climate areas with a high occurrence of such events, which contrasts with the lack of strategies needed to overcome such constraints in milder regions (derived from predictions by [37]). This rationale could as well explain the dominance of interstitial and burrower taxa in *Harsh Summer Temperate* wetlands, which are more prone to severe drought, and of attached/fixed taxa in *Mild Summer Temperate* ones, where traits favoring resistance against major disturbances should be less favored.

Furthermore, our meta-analysis showed that the patterns of trait richness and composition observed in the case study could be generalized to a global scale: in both the Nearctic and Western Palearctic, trait composition and richness differed significantly among climatic regions. In both ecozones, trait richness was highest in *Mild Summer Temperate* wetlands, and decreased towards both *Arid* and *Cold* climates. These results appear to be consistent with those described by Bêche and Statzner [9] from USA streams: although they found that environmental factors were more important than pure spatial factors (latitude, longitude) in structuring trait richness across macroinvertebrate communities, global trait richness was generally higher in the eastern and western USA and lower in the central region, and it decreased when moving from South (25–30° N) to Northern latitudes (45–50° N). 

Overall, we provide different sources of evidence that place environmental filtering as a primary influence on community assembly of seasonal wetland invertebrates. Environmental filtering (i.e. niche filtering) and competition have been long described as two non-mutually-exclusive mechanisms that theoretically govern the phylogenetic structure of many communities [3,24,38,39]. Within this context, whereas at evolutionary scales niche filtering may cause phylogenetic attraction (i.e., underdispersion, or higher relatedness than expected by chance), competition may cause phylogenetic repulsion (i.e., overdispersion, or lower relatedness than expected by chance), with the observed structure of local communities being a balance between these antagonistic forces. In freshwater habitats, local processes are known to shape the assembly rules of macroinvertebrate communities undergoing succession [40–42], similarly to what has been described for macrophytes [43], zooplankton [44], or amphibians [45]. Nevertheless, biotic interactions have been seldom considered major mechanisms controlling invertebrate biodiversity, either in streams [5,9] or in wetlands [6]. 

Although we do not reject more complex frameworks (e.g. the coexistence theory, [4]), our study suggests that focusing on environmental filters may be necessary to improve the mechanistic understanding of the macroinvertebrate communities inhabiting ephemeral lentic habitats. Therefore, we conclude that the heuristic framework of landscape filters and species traits [7] might also apply to ephemeral lentic habitats, since the ecological constraint that the dry phase represents for invertebrates may be stronger or weaker depending on the climate under which the temporary wetland they inhabit is located.

### Climate-dependent relatedness: current patterns and implications for future scenarios

Some studies have described cases in which co-occurring vertebrates [23,46] or invertebrates [9,36] are more similar to one another than would be expected by chance (a phenomenon known as self-organized similarity, see 47). This trend, associated to environmental filtering, was also observed in our study: traits present in local communities were in general non-random associations of the regional pool of available traits, as were the taxa “carrying” these traits. However, this non-randomness was not constant but climate-dependent, increasing together with the filtering effect of this factor (i.e., increasing from *Mild Summer Temperate* towards *Arid* and *Cold* climate wetlands). Thus, we suggest there is a link between the harshness of the climate under which a given wetland is located and the functional and phylogenetic (i.e. taxonomic) similarity of the community it hosts. Although phylogenetic clustering had already been described from floral and faunal communities inhabiting arid Steppe-land temporary wetlands (when compared to communities inhabiting close but milder Mediterranean wetlands, [17]), the current study confirms the robustness of this pattern at a much larger scale. Random clustering was, however, the dominant trend, indicating that in most cases local communities could be explained as random combinations from the taxa present at the climate-level scale. 

These results are particularly timely for two reasons. On one hand, large-scale biodiversity patterns and their causes have been described for several groups of organisms [48], including vascular plants [49,50] and vertebrates [51–53], but this topic remains poorly developed for freshwater invertebrates. Even studies that addressed these groups have focused, to date, on taxonomic rather than functional richness (but see 9,10). This fact still represents a limitation, since from an ecosystem perspective, functional characteristics of organisms can be more important than their taxonomic identity [54–56].

On the other hand, our findings on climate-dependent relatedness may also be useful in the light of ongoing climate change. Studies using ‘Space-for-time’ substitution approaches have so far contrasted faunal communities of streams at different geothermal temperatures [57,58] or among different ecoregions [10,59], yet lentic habitats have been seldom considered (but see 60,61). Climatic models predict that B, C, D and E Köppen climate types will successively shift to the north in the Northern Hemisphere (http://koeppen-geiger.vu -wien.ac.at, [62]). In particular, it is globally expected that coverage of B (Dry) climates (+2.68%) will increase, coverage of D (Cold) (-2.14%) and E (Polar) climates (–4.11%) will decrease, and C (Temperate) climates, currently at 15.20%, are expected to minimally change (+0.53%) assuming the IPCC’s A1FI emission scenario for the period 2076–2100. Therefore, if these predictions are accurate we may be able to anticipate some shifts in trait richness and relatedness trends in seasonal wetlands of the Northern Hemisphere. 

Within this context our results imply that, in terms of trait richness, the loss of Cold wetlands could be compensated in the future with the increase of Dry wetlands. However, the phylogenetic (taxonomic) relatedness analysis (Figure 7) implies that this replacement would cause an increase in clustering of local communities: not only is Delta+ slightly lower in Dry (B) than in Cold (D) wetlands in both ecozones, but the proportion of clustered sites is much higher in Dry than in Cold wetlands (33 % of B sites vs. 22 % of D sites in the Nearctic; 50 % of B vs. 11 % of D sites in the Western Palearctic). This could mean that by the end of the century a similar number of species, but more closely related, could develop a similar number of functions in the “new” Dry (B) seasonal wetlands with respects to the Continental (D) wetlands being lost. Nevertheless, any direct replacement between D and C wetland communities, and between C and B ones, assumes that species are able to shift their distribution ranges in relatively short periods of time (e.g., through active migration, or passive dispersal strategies). However, the few long-term (≥20 years) studies on recent climate change suggest that climatic debts (i.e., the inability to track climate change at large spatial scales) are common even in groups with high dispersal abilities, such as birds and butterflies [63]. In the United Kingdom, where long-term biomonitoring has provided data for freshwater groups (e.g., odonates, aquatic bugs, and amphibians), assessments over ca. 25 years suggest that the responses of these aquatic taxa can mirror, or even exceed, the responses to climate change described by plants, birds or butterflies [18]. Therefore, if climate change is rapid enough so that the “new” equilibrial conditions between species and their bioclimatic envelopes cannot keep pace, we suggest the observed increases in relatedness may present far reaching impacts on the invertebrate biodiversity of temporary wetlands. 

## Supporting Information

File S1 **List of sources used for the meta-analysis and associated details (Table A), and procedure followed to merge the categories and biological traits of the selected data bases (Table B).**
(DOC)
