# Supplementary material for: Functional and Phylogenetic Relatedness in Temporary Wetland Invertebrates: Current Macroecological Patterns and Implications for Future Climatic Change Scenarios
Source: PLoS One. 2013 Nov 28;8(11):e81739. doi: 10.1371/journal.pone.0081739 (PMC3842952; doi:10.1371/journal.pone.0081739)
Supplement: File S1 — List of sources used for the meta-analysis and associated details (Table A), and procedure followed to merge the categories and biological traits of the selected data bases (Table B). (DOC) [file pone.0081739.s001.doc]

**Table A.** List of sources used for the meta-analysis and associated details (N.D. = Not Determined).

| **# Source** | **Reference** | **Ecozone** | **Location** | **Köppen climate** | **# Sites** | **# Visits considered** |
| --- | --- | --- | --- | --- | --- | --- |
| 1 | [1] | NE | Mississippi (USA) | Cf | 3 | 32 |
| 2 | [2] | NE | South Carolina (USA) | Cf | 1 | 2 |
| 3 | [3] | NE | Iowa (USA) | D | 1 | 3 |
| 4 | [4] | NE | California (USA) | Cs | 1 | 4 |
| 5 | [5] | NE | Arizona (USA) | Cs | 6 | 1 |
| 6 | [6] | NE | California (USA) | Cs | 5 | 3 |
| 7 | [7] | NE | Texas (USA) | B | 2 | 16 |
| 8 | [8] | NE | Florida (USA) | Cf | 1 | 1 |
| 9 | [9] | NE | California (USA) | Cs | 1 | 3 |
| 10 | [9] | NE | Nevada (USA) | B | 3 | 1 |
| 11 | [9] | NE | Arizona (USA) | Cs | 1 | 2 |
| 12 | [9] | NE | Utah (USA) | D, Cs, B | 13 | 1-3 |
| 13 | [9] | NE | Colorado (USA) | D | 5 | 2 |
| 14 | [10] | NE | Georgia (USA) | Cf | 9 | 13 |
| 15 | [11] | NE | New Mexico (USA) | B | 1 | 14 |
| 16 | [12] | NE | California (USA) | Cs | 6 | 3 |
| 17 | [13] | NE | Wisconsin (USA) | D | 6 | N.D. |
| 18 | [14] | NE | New Mexico, Texas (USA) | B | 4 | 3 |
| 19 | This study | NE | Georgia (USA) | Cf | 8 | 3 |
| 20 | [15] | NE | Nebraska (USA) | D | 3 | 2 |
| 21 | [16] | NE | Pennsylvania (USA) | D | 2 | N.D. |
| 22 | [17] | NE | Colorado (USA) | D | 35 | N.D. |
| 23 | [18] | WP | Italy | Cs | 1 | 3 |
| 24 | [19] | WP | United Kingdom | Cf | 4 | 6 |
| 25 | [20] | WP | United Kingdom | Cf | 1 | 1 |
| 26 | [21] | WP | Catalonia, Spain | Cf, Cs, B | 15 | 1 |
| 27 | [22] | WP | Italy | Cs | 3 | ? |
| 28 | [23] | WP | Corsica, France | Cs | 1 | 2 |
| 29 | [24] | WP | Italy | Cs | 7 | 3 |
| 30 | [25] | WP | Finland | D | 1 | 1 |
| 31 | [26] | WP | Germany | D | 7 | N.D. |
| 32 | [27] | WP | Sweden | D | 8 | 1 |
| 33 | Boix et al. (unpublished data) | WP | Catalonia, Spain | Cs, Cf | 4 | 1 |
| 34 | [28] | WP | Minorca, Spain | Cs | 13 | 2 |
| 35 | [29] | WP | Morocco | B | 12 | 8 |
| 36 | [30] | WP | Switzerland | D | 3 | 1 |
| 37 | [31] | WP | Ireland | Cf | 5 | 1 |
| 38 | This study | WP | Catalonia, Spain | Cs | 6 | 3 |
| 39 | Ruhí et al. (unpublished data) | WP | Catalonia, Spain | B | 6 | 3 |
| 40 | This study | WP | Sardinia, Italy | Cs | 6 | 3 |
| 41 | [32] | WP | Spain | Cs | 1 | 1 |
| 42 | [33] | WP | France | Cs | 4 | 36 |

**Table B. Details of the procedure followed to merge the categories and biological traits of the selected data bases [34,35]. The 52 biological traits (bolded) are those considered in the *trait matrix*.**

| **Category** | **Biological trait** | **Considered traits  from Tachet et al. [35]** | **Considered traits  from EPA [34]** | **Category and trait merging procedure** |
| --- | --- | --- | --- | --- |
| **Body size** | **Large** | 2-4 cm >4-8 cm >8 cm | > 16 mm | Merging of *Maximal potential size* [35] and *Max_body_size* [34] categories.  No traits eliminated in this category. |
|  | **Medium** | 1-2 cm | 9-16 mm |
|  | **Small** | ≤.25 cm >.25-.5 cm >.5-1 cm | < 9 mm |
| **Respiration** | **Gills** | Gills | Gills | Merging of *Respiration* [35] and *Resp* [34] categories.  “Hydrostatic vesicle” trait eliminated from Tachet et al. [35]. |
|  | **Plastron, Spiracle** | Plastron Spiracle | Plastron and spiracle |
|  | **Tegument** | Tegument | Tegument |
| **Reproduction** | **Eggs, free** | Isolated eggs, free | Eggs_cement > “No” + Eggs > “Eggs_single” | Merging of *Reproduction* [35] with *Ovipos_behav_prim, Ovipos_behav_sec, Eggs_cement, Eggs_single, Eggs_1mass,* and *Eggs_multiple_batch* [34] categories.  In EPA [34], merging of “primary” and “secondary” strategies for each trait, and elimination of “other”, and Ovipos_behav “Bank soil”, “Bottom sediments”, “Floating debris”, “Free-floating”, “On/under stones (submerged)” traits. “Ovoviviparity” and “Asexual reproduction” traits eliminated in Tachet et al. [35]. |
|  | **Eggs, cemented** | Isolated eggs, cemented | Eggs_cement > “Yes” + Eggs > “Eggs_single” |
|  | **Clutches, cemented or fixed** | Clutches, cemented or fixed | Eggs_cement > “Yes” + Eggs > “Eggs_1mass,” “Eggs_multiple_batch” |
|  | **Clutches, free** | Clutches, free | Eggs_cement > “Yes” + Eggs > “Eggs_1mass”, “Eggs_multiple_batch” |
|  | **Clutches, in vegetation** | Clutches, in vegetation | Ovipos_behav > “Algal mats”, “In moss/macrophytes (submerged)”, “In wet wood” + Eggs > “Eggs_1mass”, “Eggs_multiple_batch” |
|  | **Clutches, terrestrial** | Clutches, terrestrial | Ovipos_behav > “Overhanging substrate (dry)” + Eggs > “Eggs_1mass”, “Eggs_multiple_batch” |

(cont.)

| **Category** | **Biological trait** | **Considered traits  from Tachet et al. [35]** | **Considered traits  from EPA [34]** | **Category and trait merging procedure** |
| --- | --- | --- | --- | --- |
| **Feeding mode** | **Filter-feeder** | Filter-feeder | Collector-filterer | Merging of *Feeding Habits* [35] with *Feed_prim_abbrev* and *Feed_mode_sec* [34] categories.  In EPA [34], merging of “primary” and “secondary” strategies for each trait, and elimination of “other”. |
|  | **Collector** | Deposit feeder | Collector-gatherer |
|  | **Scraper** | Scraper | Herbivore (scraper)  Scraper/grazer |
|  | **Shredder** | Shredder | Shredder |
|  | **Piercer** | Piercer | Piercer herbivore |
|  | **Predator** | Predator | Predator |
|  | **Parasite** | Parasite | Parasite |
| **Habitat** | **Attached/fixed** | Temporarily attached, permanently attached | Attached/fixed | Merging of *Locomotion and substrate relation* [35] with *Habit_prim* and *Habit_sec* [34] categories.  In EPA [34], merging of “primary” and “secondary” strategies for each trait, and elimination of “planktonic” and “diver” traits. In Tachet et al. [35], elimination of “flier” trait. |
|  | **Burrower** | Burrower | Burrower |
|  | **Skater** | Surface swimmer | Skater |
|  | **Swimmer** | Full water swimmer | Swimmer |
|  | **Interstitial** | Interstitial | Sprawler |
|  | **Other** | Crawler | Other  (Climber,clinger, crawler) |
| **Microhabitat** | **Flags/boulders/ cobbles/pebbles** | Flags/boulders/ cobbles/pebbles | Rocks  Boulder | Merging of *Substrate (preferendum)* [35] with *Microhab* [34] categories.  In EPA [34], elimination of “pelagic” trait; in Tachet et al. [35], elimination of “twigs/roots” and “mud” traits. |
|  | **Gravel** | Gravel | Gravel |
|  | **Sand** | Sand | Sand |
|  | **Silt** | Silt | Silt |
|  | **Macrophytes** | Macrophytes | Plants |
|  | **Microphytes** | Microphytes | Phyto  Algae |
|  | **Detritus/litter** | Organic detritus/litter | LWD (large woody debris)  Detritus |

(cont.)

| **Category** | **Biological trait** | **Considered traits  from Tachet et al. [35]** | **Considered traits  from EPA [34]** | **Category and trait merging procedure** |
| --- | --- | --- | --- | --- |
| **Exit temporarily** | **Absent** | Aquatic passive  Aerial passive | Absent | Merging of *Dispersal* [35] with *Exit temporarily* [34] categories.  Elimination of “Aquatic active” trait in Tachet et al. [35]. |
|  | **Present** | Aerial active | Present |
| **Number of aquatic stages** | **1 (larva/nymph only)** | 1 (larva/nymph  only) | 1 (larvae/nymph  only) | Merging of *Aquatic stages* [35] with *Aquatic stages* [34] categories.  No traits eliminated in this category. |
|  | **2 (egg, larva/nymph)** | 2 (egg,  larva/nymph) | 2 (egg,  larvae/nymph) |
|  | **3 (egg, larva/nymph, adult; or egg, larva, pupa)** | 3 (egg,  larva/nymph,  adult; or egg, larva,  pupa) | 3 (egg,  larva/nymph,  adult; or egg, larva, pupa) |
|  | **4 (egg, larva, pupa, adult)** | 4 (egg, larva,  pupa, adult) | 4 (egg, larva,  pupa, adult |
| **Voltinism** | **< 1** | < 1 | Semivoltine | Merging of *Potential number of cycles per year* [35] with *Voltinism* [34] categories.  No traits eliminated in this category. |
|  | **1** | 1 | Univoltine |
|  | **> 1** | >1 | Bi_multivoltine |
| **Trophic preferendum** | **Oligotrophic** | Oligotrophic | O2 high | Merging of *Trophic status (preferendum)* [35] with *O2_high, O2_normal,* and *O2_low* [34] categories.  No traits eliminated in this category. |
|  | **Mesotrophic** | Mesotrophic | O2 normal |
|  | **Eutrophic** | Eutrophic | O2 low |

(cont.)

| **Category** | **Biological trait** | **Considered traits  from Tachet et al. [35]** | **Considered traits  from EPA [34]** | **Category and trait merging procedure** |
| --- | --- | --- | --- | --- |
| **pH preferendum** | **Acidic** | ≤ 4  > 4 - 4.5 | pH_acidic | Merging of *pH (preferendum)* [35] with *pH_acidic, pH_normal,* and *pH_alkaline* [34] categories.  No traits eliminated in this category. |
|  | **Normal** | > 4.5 – 5  5 - 5.5 | pH_normal |
|  | **Alkaline** | 5.5-6  > 6 | pH_alkaline |
| **Salinity preferendum** | **Fresh waters** | Fresh water | Salin_fresh | Merging of *Salinity (preferendum)* [35] with *Salin* [34] categories.  “Salin_salt” trait was eliminated from EPA [34]. |
|  | **Brackish waters** | Brackish water | Salin_brackish |
| **Temperature preferendum** | **Psychrophilic** | Psychrophilic | Cold stenothermal | Merging of *Temperature* [35] with *Thermal_pref* [34] categories.  “No strong preference” trait was eliminated from EPA [34]. |
|  | **Thermophilic** | Thermophilic | Hot euthermal |
|  | **Eurythermic** | Eurythermic | Cold-cool eurythermal Warm eurythermal |

**References Tables A & B in File S1**

1. Bonner LA, Diehl WJ, Altig R (1997) Physical, chemical and biological dynamics of five temporary dystrophic forest pools in Central Mississippi. Hydrobiologia 353: 77-89.

2. Braccia A, Batzer D (2001) Invertebrates associated with woody debris in a Southeastern U.S. forested floodplain wetland. Wetlands 21: 18-31.

3. Christensen J, Crumpton W (2010) Wetland invertebrate community responses to varying emergent litter in a prairie pothole emergent marsh. Wetlands 30: 1031-1043.

4. De Szalay FA, Resh VH (2000) Factors influencing macroinvertebrate colonization of seasonal wetlands: responses to emergent plant cover. Freshw Biol 45: 295-308.

5. Graham TB (2002) Survey of aquatic macroinvertebrates and amphibians at Wupatki National Monument, Arizona, USA: An evaluation of selected factors affecting species richness in ephemeral pools. Hydrobiologia 486: 215-224.

6. Marchetti MP, Garr M, Smith ANH (2010) Evaluating wetland restoration success using aquatic macroinvertebrate assemblages in the Sacramento Valley, California. Rest Ecol 18: 457-466.

7. Merickel FW, Wangberg JK (1981) Species composition and diversity of macroinvertebrates in two playa lakes on the Southern High Plains, Texas. Southwest Nat 26: 153-158.

8. Merritt RW, Higgins MJ, Cummins KW, Vandeneeden B (1999) The Kissimmee river–riparian marsh ecosystem, Florida. In: Batzer DP, Rader RD, Wissinger SA, editors. Invertebrates in freshwater wetlands of North America: ecology and management. New York: John Wiley and Sons. pp. 55-79.

9. BLM/USU (pers. comm.) BLM/USU (US Bureau of Land Management/Utah State University) National Aquatic Monitoring Center, Logan, UT.

10. Reese EG, Batzer DP (2007) Do invertebrate communities in floodplains change predictably along a river's length? Freshw Biol 52: 226-239.

11. Richardson G, Ward CR, Huddleston EW (1972) Aquatic macroinvertebrates of the playa. Logan, UT: U.S. International Biological Program, Desert Biome. 30 p.

12. Rogers C (1998) Aquatic macroinvertebrate occurrences and population trends in constructed and natural vernal pools in Folsom, California. In: Witham CW, Bauder ET, Belk D, Ferren Jr. WR, Ornduff R, editors. Ecology, Conservation, and Management of Vernal Pool Ecosystems – Proceedings from a 1996 Conference. Sacramento, CA: California Native Plant Society. pp. 224-235.

13. Schneider DW, Frost TM (1996) Habitat duration and community structure in temporary ponds. J North Am Benthol Soc 15: 64-86.

14. Sublette JE, Sublette MS (1967) The limnology of playa lakes on the Llano Estacado, New Mexico and Texas. Southwest Nat 12: 369-406.

15. Whiles M, Goldowitz B (2005) Macroinvertebrate communities in central Platte River wetlands: Patterns across a hydrologic gradient. Wetlands 25: 462-472.

16. Wissinger SA, Gallagher LJ (1999) Beaver pond wetlands in Northwestern Pennsylvania. Modes of colonization and sucesssion after drought. In: Batzer DP, Rader RD, Wissinger SA, editors. Invertebrates in freshwater wetlands of North America: ecology and management. New York: John Wiley and Sons. pp. 333-362.

17. Wissinger SA, Bohonak AJ, Whiteman HH, Brown WS (1999) Subalpine wetlands in Colorado. Habitat permanence, salamander predation, and invertebrate communities. In: Batzer DP, Rader RD, Wissinger SA, editors. Invertebrates in freshwater wetlands of North America: ecology and management. New York: John Wiley and Sons. pp. 757-790.

18. Bazzanti M, Baldoni S, Seminara M (1996) Invertebrate macrofauna of a temporary pond in Central Italy: composition, community parameters and temporal succession. Archiv Hydrobiol 137: 77-94.

19. Bevercombe A, Cox N, Thomas M, Young J (1973) Studies of the invertebrate fauna of a wet slack in a sand dune system. Archiv Hydrobiol 71: 487-516.

20. Blackstock TH, Duigan CA, Stevens DP, Yeo MJM (1993) Case studies and reviews. Vegetation zonation and invertebrate fauna in Pant–y–llyn, an unusual seasonal lake in South Wales, UK. Aquat Conserv: Mar Freshwat Ecosyst 3: 253-268.

21. Boix D, Gascón S, Sala J, Badosa A, Brucet S, et al. (2008) Patterns of composition and species richness of crustaceans and aquatic insects along environmental gradients in Mediterranean water bodies. Hydrobiologia 597: 53-69.

22. Cianficconi F, Moretti G, Pirisinu Q, Tucciarelli F (1976) Composizione sistematica delle comunità acquatiche del settore meridionale dei Monti Sibillini, con considerazioni zoogeografiche. Lav Soc It Biogeogr 6: 479-524.

23. Culioli J, Foata J, Mori C, Orsini A, Marchand B (2006) Temporal succession of the macroinvertebrate fauna in a Corsican temporary pond. Vie et milieu 56: 215-221.

24. Della Bella V (2005) Composizione tassonomica, organizzazione funzionale e struttura in taglia della macrofauna a invertebrati di biotopi temporanei e permanenti del litorale tirrenico. Roma: Università di Roma "La Sapienza". 150 p.

25. Hirvenoja M (2002) The fauna in two cold springs and in an epirhithral pool in southern Finland. Sahlbergia 7: 7-25.

26. Kramer H (1964) Okologische untersuchungen an temporaren tumpeln des bonner kottenforstes. Decheniana 117: 53-132.

27. Liungman M (2008) Linsräka i Stockholms län. En inventering av bottenfauna i efemära vatten på nio platser i Stockholms län 2007. Stockholm: Länsstyrelsens I Stockholms län. 39 p.

28. Boix D, Ruhí A, Sala J, Gascón S, Compte J, et al. (2009) Aportació al coneixement de les associacions de macrofauna de les basses temporanies de Menorca. Projecte Life Basses, Life05/Nat/ES/000058. Maó: Consell Insular de Menorca. 21 p.

29. Metge G (1986) Etude des écosystèmes hydromorphes (daya, merja) de la Méséta occidentale marocaine: typologie et synthèse cartographique à objectif sanitaire appliquée aux populations d'*Anopheles labranchiae* (Falleroni, 1926), (Diptera, Culicidae, Anophelinae). Aix-en-Provence: Université de Droit, d’Economie et Sciences d’Aix-Marseille. 280 p.

30. Oertli B, Indermuehle N, Angélibert S, Hinden H, Stoll A (2008) Macroinvertebrate assemblages in 25 high alpine ponds of the Swiss National Park (Cirque of Macun) and relation to environmental variables. Hydrobiologia 597: 29-41.

31. Porst G, Irvine K (2009) Distinctiveness of macroinvertebrate communities in turloughs (temporary ponds) and their response to environmental variables. Aquat Conserv: Mar Freshwat Ecosyst 19: 456-465.

32. Sahuquillo M, Poquet J, Rueda J, Miracle M (2007) Macroinvertebrate communities in sediment and plants in coastal Mediterranean water bodies (Central Iberian Peninsula). Ann Limnol – Int J Lim 43: 117-130.

33. Terzian E (1979) Ecologie des mares temporaires de l'Isoetion dans la Crau et l'Esterel (France). Aix-en-Provence: Université de Droit, d’Economie et Sciences d’Aix-Marseille. 210 p.

34. U.S. EPA. (2012) Freshwater Biological Traits Database. Washington, DC: United States Environmental Protection Agency. Available: <http://www.epa.gov/ncea/global/traits/>. Accessed 1 December 2012.

35. Tachet H, Richoux P, Bournaud M, Usseglio–Polatera P (2002) Invertebrés d’eau douce (2nd corrected impression). Paris: CNRS éditions. 587 p.
